# Tracheobronchial invasion by nontuberculous mycobacteria: a rare but overlooked clinical manifestation—a multicenter retrospective analysis

**DOI:** 10.3389/fcimb.2026.1872833

**Published:** 2026-07-13

**Authors:** Mianluan Pan, Yaqin Wei, Chunyuan Luo, Hairong Lin, Weiguang Lu, Yuhao Lin, Ziqing Mai, Jiehua Deng, Yanmei Huang, Haiqiong Yu, Jie Huang, Jianquan Zhang

**Affiliations:** 1 Department of Respiratory and Critical Care Medicine, The Eighth Affiliated Hospital of Sun Yat-sen University, Shenzhen, Guangdong, China; 2 Department of Tuberculosis, Chest Hospital of Guangxi Zhuang Autonomous Region, Liuzhou, Guangxi, China; 3 Department of Tuberculosis, HIV/AIDS Clinical Treatment Center of Guangxi (Nanning) and The Fourth People’s Hospital of Nanning, Nanning, Guangxi, China

**Keywords:** anti-IFN-γ autoantibody, bronchoscopy, diagnostic delay, *Mycobacterium colombiense*, nontuberculous mycobacteria, tracheobronchial disease

## Abstract

**Background:**

Nontuberculous mycobacteria (NTM) can disseminate and infect various organs throughout the body. However, whether NTM can infect tracheobronchial tissue is rarely reported. This study aimed to address the knowledge gap regarding the epidemiological, demographic, and clinical characteristics of patients with tracheobronchial NTM infection.

**Methods:**

In this multicenter retrospective cohort study, clinical, demographic, microbiological, and radiological data from hospitalized patients with tracheobronchial NTM infections from January 2015 to May 2025 were collected and analyzed descriptively.

**Results:**

Twenty-nine patients (2.3%) were included, and all the patients presented with disseminated NTM infection. Seventeen patients had comorbidities, including 5 with acquired immunodeficiency syndrome and 1 with anti-interferon-γ autoantibody syndrome. Median diagnostic delay was 130 days, and 89.7% of the patients were initially misdiagnosed with tuberculosis or malignancy. The most common symptoms were cough, expectoration, anemia, fever, weight loss, skin lesions, and bone pain. Chest CT revealed nodules, patchy opacities, mass-like shadows, and bronchial stenosis, with or without hilar/mediastinal lymphadenopathy, whereas osteolytic bone destruction was evident in 11 patients. The most common features of bronchoscopy were intraluminal masses/neoplasms/nodules. Metagenomic next-generation sequencing (mNGS) of BALF (n=12) demonstrated 100% positivity, outperforming BALF culture (46.2%, 12/26) and sputum culture (39.3%, 11/28). *Mycobacterium colombiense* accounted for 24.1% of cases. With respect to therapeutic management, 27 patients received systemic antimicrobial therapy, while 2 did not receive specific anti-N™ treatment. One patient underwent combined endoscopic resection. Overall, 23 patients (79.3%) achieved improvement or cure, 5 showed disease progression, 1 experienced relapse, and 1 died.

**Conclusions:**

Tracheobronchial NTM infection is rare but clinically significant, often occurring in the context of disseminated disease with pulmonary involvement. Immunocompromised hosts, particularly those with AIDS or anti–IFN-γ autoantibody syndrome, are highly susceptible. Bronchoscopy typically reveals mass lesions causing luminal stenosis or occlusion. In this cohort, *M. colombiense* was the most frequently isolated NTM species. Early bronchoscopy, mNGS-based pathogen detection, and timely systemic or endoscopic intervention should be considered to prevent irreversible airway stenosis. Further studies are needed to validate optimal treatment strategies.

**Clinical Trial Registration:**

https://www.ClinicalTrials.gov, identifier NCT07377864.

## Introduction

Nontuberculous mycobacterial pulmonary disease (NTM-PD) is a chronic respiratory infection caused by mycobacteria other than the *Mycobacterium tuberculosis* complex and *Mycobacterium leprae* (Johansen et al., 2020). NTM-PD is the most common clinical presentation. Beyond the lungs, NTM can disseminate to various extrapulmonary sites, including the skin and soft tissues (Gonzalez-Santiago and Drage, 2015; Ford et al., 2023), lymph nodes, bone marrow/bloodstream, bones/joints (Bi et al., 2015; Tang et al., 2021; Tang et al., 2023), central nervous system (Meena et al., 2023), liver and spleen (Mahmood et al., 2018), muscles (Gundavda et al., 2017), heart valves (Meena et al., 2024), and eyes (Kheir et al., 2015; Kim et al., 2020). However, NTM direct tracheobronchial tree invasion remains poorly characterized and is often buried within case reports of “endobronchial tuberculosis” misdiagnoses (Koh et al., 2006). To date, available evidence has been limited to isolated case reports and small single-center series (Kröner et al., 2015; Bell et al., 2023; Lau et al., 2023; Liang et al., 2025), with scarce data on bronchoscopic features, species distribution, and treatment outcomes. Notably, no multicenter epidemiological study has systematically characterized the demographic, clinical, and microbiological profiles of this entity. South China represents a high-burden region for NTM disease; however, multicenter studies focusing on airway involvement are lacking.

This multicenter retrospective analysis bridges this gap by providing a systematic evaluation of tracheobronchial NTM infection across three tertiary centers in South China. Specifically, we sought to (1) characterize the bronchoscopic, radiological, and microbiologic features of NTM tracheobronchial invasion; (2) identify species-specific and host-specific phenotypes, particularly *M. colombiense* and anti-IFN-γ autoantibody syndrome; (3) assess the impact of mNGS implementation on diagnostic efficiency; and (4) evaluate treatment outcomes, specifically testing the hypothesis that advanced structural damage is reversible with targeted therapy.

## Methods

### Study design and participating centers

We collected data from 29 patients with NTM disease involving the trachea and/or bronchi (segmental bronchi and above) across three tertiary hospitals in southern China between January 1, 2015, and May 1, 2025. We analyzed their epidemiological characteristics, clinical features, laboratory test results, radiological findings, and treatment outcomes. The clinical trial registration number for the study is NCT07377864 (www.ClinicalTrials.gov). All study procedures strictly adhered to the principles of the Declaration of Helsinki. This study was approved by the institutional ethics committee of the Eighth Affiliated Hospital of Sun Yat-Sen University (2025-184-01). The informed consent requirement was waived because of the nature of the study.

The inclusion criteria were (1) comprehensive bronchoscopy examination revealing direct tracheobronchial invasion, defined as the presence of mucosal abnormalities (e.g., ulceration, nodularity, a mass, thickening, erosion, or necrosis) that involve the trachea, main bronchi, or lobar/segmental bronchi, in the absence of isolated extrinsic airway compression on chest CT or endoscopic findings; airway involvement (trachea, main bronchi, or lobar/segmental bronchi); (2) NTM infection confirmed in respiratory specimens (sputum, BALF, bronchial mucosal tissue, or lung tissue obtained via BALF collection, bronchial biopsy, transbronchial lung biopsy, percutaneous lung puncture, or EBUS-TBNA); and (3) a positive confirmatory test (culture, fluorescence PCR probe melting curve, DNA microarray, histopathology, or mNGS).

The exclusion criteria were (1) NTM disease without clinical evidence of respiratory system infection; (2) respiratory symptoms without positive NTM results from respiratory specimens; (3) pulmonary parenchymal involvement with identified NTM infection but no evidence of airway lesions on bronchoscopy; (4) prior bronchoscopy or airway intervention; and (5) incomplete medical records.

Outcome definitions followed predefined criteria (Azekawa et al., 2026): Cure (resolution of all symptoms/lesions posttreatment); improvement (partial resolution of symptoms/lesions or ongoing treatment); non improvement (failure to meet cure or improvement criteria); recurrence (culture reconversion in ≥2 samples posttreatment completion); and death (all-cause mortality following diagnosis).

### Statistical analysis

Continuous variables were assessed for normality via the Shapiro–Wilk test. As the data were not normally distributed, they were presented as medians with interquartile ranges (IQRs). Categorical variables were summarized as frequencies and percentages.

Group comparisons for continuous variables were performed by using the Mann–Whitney U test. Categorical variables were compared by using Fisher’s exact test, which is appropriate for small sample sizes and when expected cell frequencies are less than five.

To identify factors that were independently associated with the diagnostic delay, bronchial obstruction, and bone destruction, we first performed univariate logistic regression analyses for each potential predictor. Variables with a P value <0.10 in univariate analysis, together with clinically relevant variables (age, sex, and symptom duration), were entered into multivariate logistic regression models.

Given the limited sample size (n=29) and the potential for sparse data bias or complete separation, we applied Firth’s penalized maximum likelihood logistic regression by using the logistf package in R. This method reduces first-order bias in parameter estimates and provides more reliable inference in small-sample settings.

For patients with mixed NTM infections (n=2), classification in the primary analysis was based on the clinically predominant species. A sensitivity analysis, excluding these patients, was performed to verify the robustness of the findings.

Internal validation of the multivariate models was conducted by using bootstrap resampling with 1,000 iterations to estimate optimism-corrected confidence intervals.

All statistical tests were two sided, and a *P* value <0.05 was considered to indicate statistical significance. Statistical analyses were performed by using the R software (version 4.3.1; R Foundation for Statistical Computing, Vienna, Austria) with the following packages: logistf, boot, and ggplot2.

## Results

### Study population and baseline characteristics

**Figure 1:**
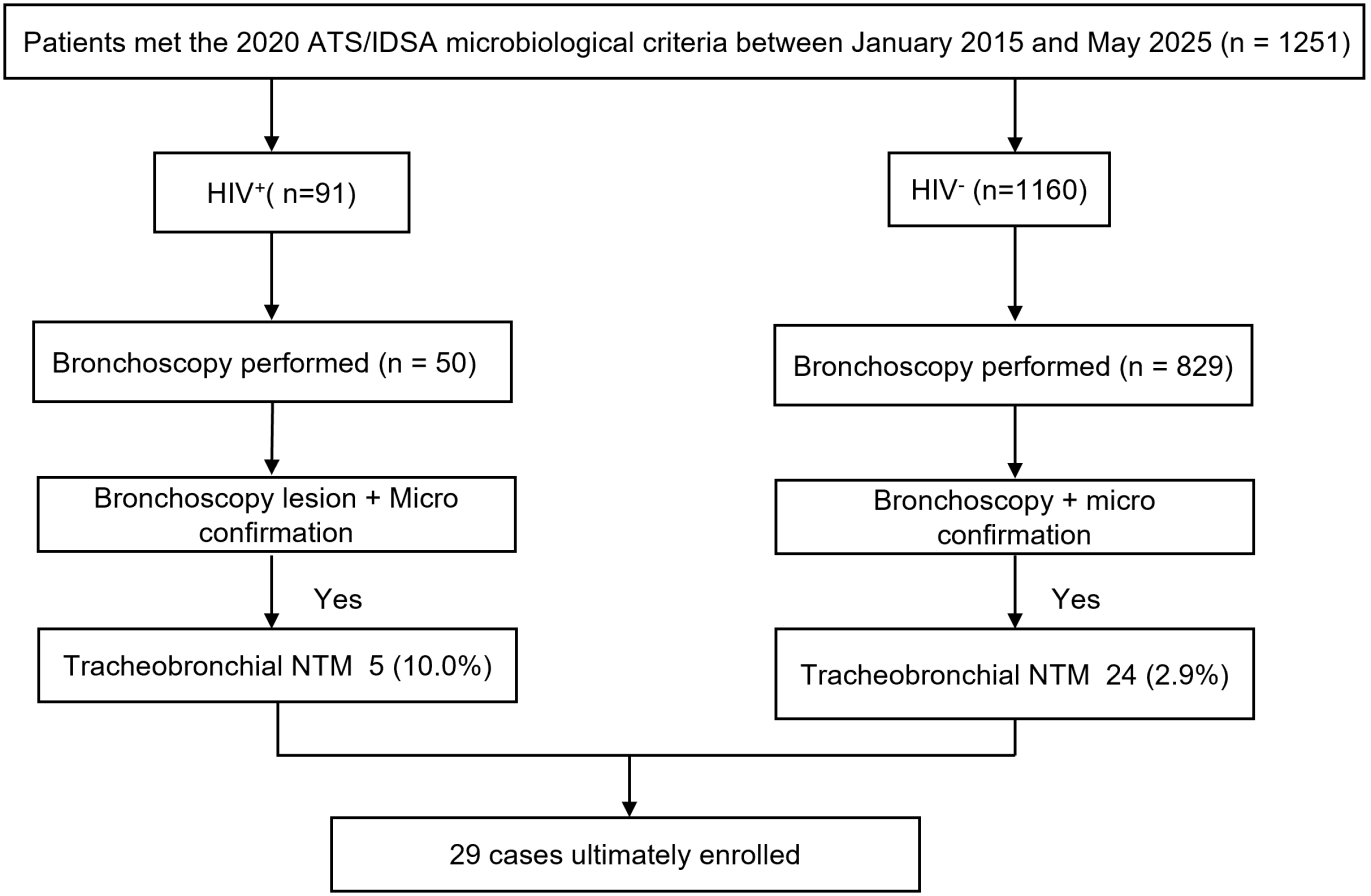
Study population. ATS/IDSA, American Thoracic Society/Infectious Diseases Society of America; HIV, human immunodeficiency virus; NTM, nontuberculous mycobacteria.

During the study period, 1,251 patients with NTM disease were screened, of whom 29 (2.3%) met the inclusion criteria for tracheobronchial NTM infection (**Figure 1**). As shown in **Table 1**, the cohort was predominantly middle aged (median age: 46.5 years), with a slight male predominance (55.2%). Immunocompromised status was common, with AIDS (17.2%) and anti–IFN-γ autoantibody syndrome (3.4%) being the most notable predisposing conditions. A substantial diagnostic delay (median 130 days) and a high misdiagnosis rate (82.8%) were observed, with pulmonary tuberculosis (65.5%) and lung cancer (17.2%) being the most common initial diagnoses.

**Table 1:** Baseline characteristics of 29 patients with NTM tracheobronchial invasion.

Characteristic	Value
Demographics	
Age, y	46.5 (40.5–54.0)
Female	13 (44.8)
Male	16 (55.2)
BMI, kg/m²	20.2(19.4–22.2)
Comorbidities	
AIDS	5 (17.2)
Diabetes mellitus	2 (6.9)
Anti-IFN-γ autoantibody syndrome	1 (3.4)
Prior pulmonary tuberculosis	3 (10.3)
Other malignancy	2 (6.9)
Clinical Presentation	
Cough	29(100)
Expectoration	26(89.7)
Hemoptysis	8(27.7)
Fever	12(41.4)
Weight loss	7(24.1)
Skin/soft tissue	10(34.5)
Lymphadenopathy	16(55.2)
Bone involvement	11(37.9)
Diagnostic Characteristics	
Diagnostic, d	130 (44-239)
Misdiagnosed as pulmonary tuberculosis	19(65.5)
Misdiagnosed as lung cancer	5(17.2)
Radiologic Features	
Cavitary lesion	3(10.3)
Nodular opacities	24(82.6)
Patchy shadows	23(79.3)
Hilar/mediastinal lymphadenopathy	17(58.6)
Linear opacities	13(44.8)
Pleural effusion	13(44.8)
Bronchial stenosis/obliteration	12(41.4)
Pleural thickening.	12(41.4)
Bone destruction	11(37.9)
Consolidation	8(27.6)
Pericardial effusion	6(20.7)
Mass	4(13.8)
Bronchiectasis	2(6.9)
Microbiologic Confirmation	
Sputum culture positive	11(39.3%, 11/28)
BALF culture positive	12(46.2%, 12/26)
mNGS positive	12(100)

All patients presented with cough, and the majority (89.7%) had expectoration. Extrapulmonary involvement, including lymphadenopathy (55.2%), bone lesions (37.9%), and skin/soft tissue involvement (34.5%), was frequent. Chest CT most commonly demonstrated nodular opacities (82.6%), patchy shadows (79.3%), and hilar/mediastinal lymphadenopathy (58.6%). Bronchial stenosis or obliteration was observed in 41.4% of the patients. Notably, osteolytic bone destruction was evident in 11 patients (37.9%). In one representative case, emission CT demonstrated extensive disseminated osteolytic lesions involving the skull, facial bones, spine, sternum, ribs, and long bones.

### Temporal trends in diagnostic efficiency

Stratification by diagnostic era revealed marked improvements coinciding with mNGS implementation (**Table 2**). The median diagnostic delay was significantly reduced, from 165 days in 2015–2019 to 75 days in 2020–2025 (P = 0.03), representing a 55% reduction. The proportion of patients with diagnostic delays exceeding 180 days decreased from 55.6% to 18.2% (P = 0.08). Concomitantly, mNGS use increased from 11.1% to 90.9% (P<0.001), and the median time to species identification was shortened from 35 to 14 days (P = 0.002). Empiric HRZE use was confined to the early period (22.2% vs. 0%, P = 0.10). Despite these diagnostic and microbiological advances, the misdiagnosis rate remained substantial (81.8% in 2020–2025), highlighting the ongoing challenge of recognizing this rare entity.

**Table 2:** Temporal trends in diagnostic characteristics (2015–2019 vs 2020-2025).

Characteristic	2015-2019 (n=18)	2020-2025 (n=11)	P Value
Demographics			
Age, y	48 (41-55)	45 (38-52)	0.54
Female sex	10 (55.6)	6 (54.5)	0.95
Diagnostic Metrics			
Diagnostic delay, d	165 (90-280)	75 (30-140)	0.03
Delay >180 d	10 (55.6)	2 (18.2)	0.08
Misdiagnosis rate	17 (94.4)	9 (81.8)	0.28
Microbiologic Methods			
mNGS utilization	2 (11.1)	10 (90.9)	<0.001
Time to species ID, d	35 (28-56)	14 (7-21)	0.002
Treatment Approach			
Empiric HRZE before diagnosis	4 (22.2)	0 (0)	0.1
Standard anti-NTM therapy	12 (66.7)	10 (90.9)	0.15
Outcomes			
Improved/Cured	14 (77.8)	10 (90.9)	0.37
Follow-up duration, mo	24 (12-48)	12 (6-24)	0.08

Data are presented as median (IQR) or n (%). *P* values from Mann-Whitney U test (continuous) or Fisher exact test (categorical). Bold values indicate *P* < 0.10. HRZE = isoniazid + rifampin + pyrazinamide + ethambutol; mNGS = metagenomic next-generation sequencing.

### NTM species and clinical phenotypes

Thirty-one NTM isolates were identified from the 29 patients, including two patients with mixed infections (*M. avium* complex and *M. colombiense*). The species distribution and associated clinical phenotypes are detailed in **Table 3**. *M. colombiense* infection exhibited distinct clinical features; in particular, 85.7% (6/7) of the patients with *M. colombiense* infection had bone destruction, compared with 22.7% (5/22) of those infected with otherNTMspecies (*P* = 0.015). However, differences in the neoplastic/polypoid morphology and cure rates between patients infected with *M. colombiense* and those infected with other species were not statistically significant (*P* > 0.05 for both; **Table 3**).

**Table 3:** NTM species distribution and clinical phenotypes.

Species	n (%)	Neoplastic/polypoid morphology	Bone destruction	Improved/cured
M. colombiense	7(24.1)	7 (100)	6 (85.7)	6 (85.7)
M. avium	6(20.7)	5 (83.3)	1(16.7)	6(100)
M. intracellulare	5(17.2)	2 (40.0)	2 (40.0)	5 (100)
M. avium complex	2 (6.9)	2(100)	2(100)	2(100)
M. abscessus	2(6.9)	2 (100)	1 (50.0)	2 (100)
M. kansasii	1 (3.4)	0 (0)	0 (0)	1 (100)
M. chelonae	1 (3.4)	0 (0)	0 (0)	1 (100)
M. florentinum	1 (3.4)	1 (100)	0 (0)	1 (100)
Not specified	6 (20.7)	3 (50.0)	2 (33.3)	4 (66.7)
Total	29(100)	25 (86.2)	11(37.9)	24 (82.8)

Data are presented as n (%). Comparisons across species were performed using Fisher’s exact test.

### Bronchoscopic spectrum

The bronchoscopic findings are detailed in **Table 4**. Anatomically, the left main bronchus was the most frequently involved site (34.5%), followed by the right upper lobe (31.0%) and right middle lobe (27.6%). Sixteen patients (55.2%) had two or more affected lobar or segmental bronchi. Lobar and segmental bronchi were more commonly affected than the main bronchi were. The most common morphological features were mass/polypoid lesions (79.3%), stenosis (51.7%), and nodular lesions (34.5%). Rough or friable surfaces (bleeding on contact) were observed in 41.4% of the patients, and serous secretions were the most common type (31.0%).

**Table 4:** Bronchoscopic features of NTM tracheobronchial invasion.

Feature	Category	n (%)
Anatomical Location		
	Trachea	3 (10.3)
	Carina	2 (6.9)
	Left main bronchus	10 (34.5)
	Right main bronchus	6 (20.7)
	Left upper lobe	7 (24.1)
	Left lower lobe	6 (20.7)
	Right upper lobe	9 (31.0)
	Right middle lobe*	8 (27.6)
	Right lower lobe	4 (13.8)
Morphology		
	Nodular lesions	10 (34.5)
	Mass/polypoid lesion	23 (79.3)
	Stenosis	15 (51.7)
	Obstruction/occlusion	6 (20.7)
Surface Characteristics		
	Smooth	2 (6.9)
	Rough/friable (bleeds on contact)	12 (41.4)
	Necrotic/gray-yellow exudate	5 (17.2)
Secretions		
	Serous/watery	9 (31.0)
	Mucoid	2 (6.9)
	Purulent	4 (13.8)

Data are presented as n (%). Percentages for morphologic features sum to >100% because lesions often had combined characteristics. For morphology classification: “Nodular lesion” refers to small, circumscribed mucosal elevations (<1 cm); “Mass/polypoid lesion” refers to larger, exophytic lesions (≥1 cm), which may be sessile or pedunculated.

### Anti-IFN-γ autoantibody syndrome

Patient 11, a 40-year-old male with newly identified anti-IFN-γ autoantibody syndrome, presented with disseminated *M. colombiense* infection (tracheobronchial, pulmonary, cutaneous, and nodal). Unlike immunocompetent *M. colombiense* patients with localized tumor-like masses, this patient experienced diffuse inflammatory changes with multiple stenoses. Treatment required a 5-drug combination (clarithromycin, rifampin, cefoxitin, ethambutol, and amikacin) with improvement. This case expands the AIGA phenotype to include primary airway involvement.

### Treatment heterogeneity and outcomes

Twenty-three patients (79.3%) received species-directed anti-NTM therapy after diagnosis. However, 4 patients (13.8%) were empirically treated with HRZE before NTM identification, reflecting diagnostic uncertainty during the early study period (2015–2019). Notably, Patient 25 (*M. chelonae*) achieved clinical improvement despite never receiving standard anti-NTM therapy, explained by the partial efficacy of ethambutol (contained in HRZE) and possible unrecognized fluoroquinolone use. We explicitly acknowledge this treatment heterogeneity as a limitation of the retrospective design (see **Supplementary Case Narrative**). The outcomes at the last follow-up were as follows: cure, 3.4% (1/29); improvement, 72.4% (21/29); non improvement, 17.2% (5/29); recurrence, 3.4% (1/29; Patient 16); and death, 3.4% (1/29; Patient 17).

**Figure 2:**
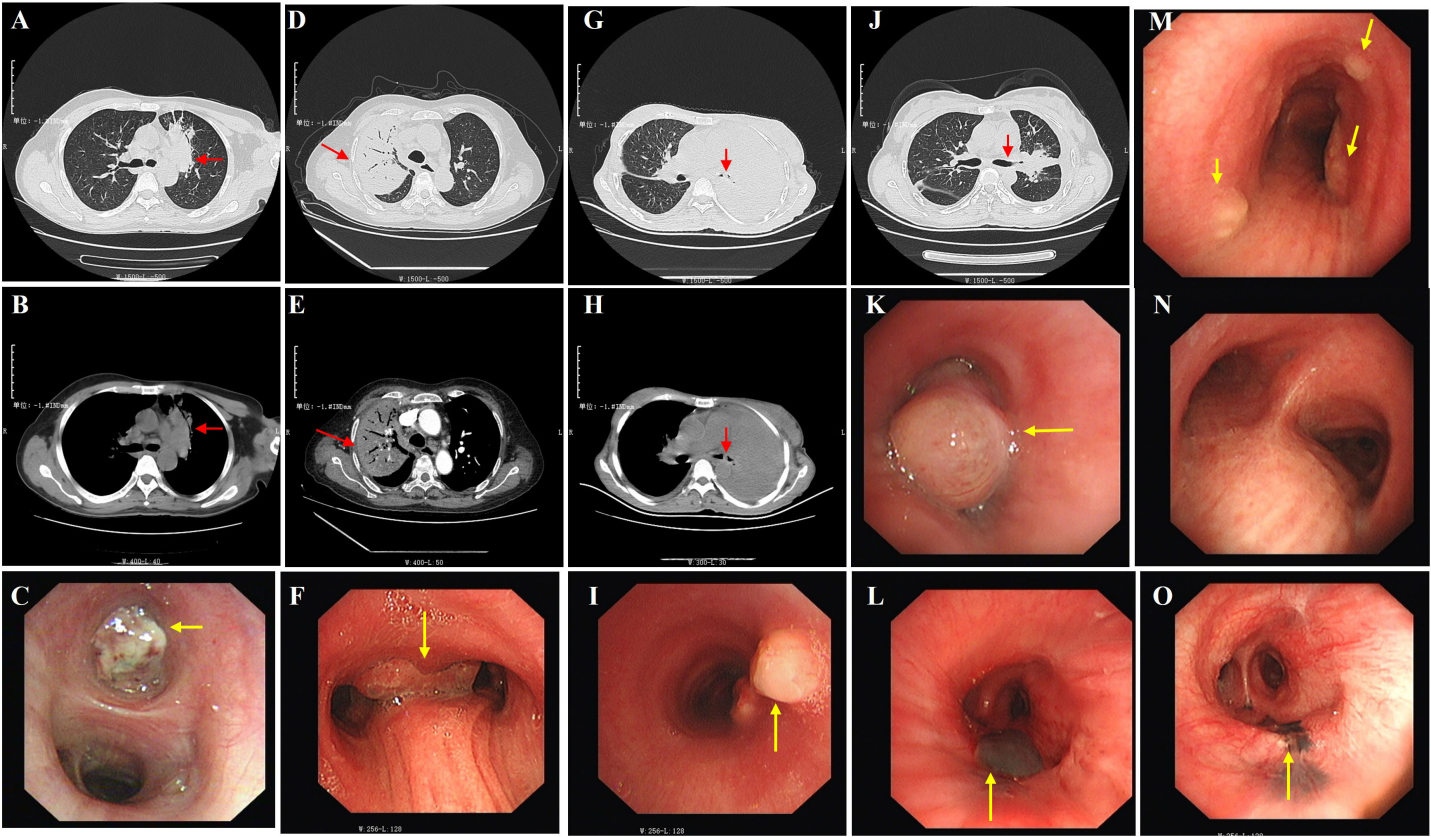
**(A, B)** Chest CT of Patient 1 revealed irregular mass-like consolidation at the left hilum with narrowing of the left main bronchus. **(C)** Bronchoscopy in Patient 1 revealed a polypoid mass completely occluding the left upper-lobe orifice, with a rough surface that bled on contact. **(D, E)** Chest CT of Patient 19 revealed left-sided thoracic cage collapse, extensive consolidation, and complete luminal occlusion, with a nodular lesion visible in the left main bronchus (arrow); **(F)** bronchoscopy demonstrated a smooth-surfaced neoplasm obstructing the same bronchus. **(G)** Follow-up chest CT after anti-NTM therapy showed left upper lobe re-expansion, a reduction in pulmonary lesions, resolution of the nodule, and a patent airway. **(H)** Bronchoscopy of Patient 2 revealed multiple, variable-sized nodular lesions diffusely distributed along the distal tracheal wall; **(I)** follow-up bronchoscopy after anti-NTM therapy revealed complete regression of these previously noted tracheal masses. **(J)** Bronchoscopy of Patient 6 revealed two discrete polypoid masses within the mid-tracheal lumen. **(K)** Bronchoscopy of Patient 16 revealed a widened carina with an irregular mass extending into both main-stem bronchi, causing bilateral proximal bronchial stenosis. **(L)** Bronchoscopy of Patient 18 revealed stenosis of the right middle-lobe orifice, with associated mucosal swelling, thickening, and surface ulceration. **(M)** Diffuse variably-sized nodular lesions in the lower trachea (Patient 2). **(N)** Complete resolution of lower tracheal lesions after 2-month anti-NTM therapy. **(O)** Resolution of the brownish lesion at the membranous portion of the right intermediate bronchus (Patient 18) following anti-NTM therapy.

Critically, Patient 19 achieved complete radiological and bronchoscopic resolution of left lung collapse with thoracic cage re-expansion after 6 months of targeted anti-N™ therapy (**Figures 2D-G**), demonstrating the reversibility of advanced structural damage. Patient 2 showed complete regression of diffuse tracheal nodules (**Figures 2H, I**).

## Discussion

We report the largest rigorously characterized cohort of patients with NTM tracheobronchial invasion to date. Three findings warrant emphasis. First, the diagnostic delay exceeded 4 months in the majority of our patients, which was frequently driven by malignancy mimicry—particularly for *M. colombiense* infection, which often presented with mass/polypoid lesions. Second, we identified adult-onset anti–IFN-γ autoantibody syndrome in patients who presented with primary airway involvement, which may expand the clinical spectrum of this underrecognized immunodeficiency. Third, our findings suggest that complete bronchial obstruction with lung collapse may be reversible in some patients following appropriate antimicrobial and endoscopic therapy, raising the possibility that irreversible airway damage might be avoidable with timely intervention. Given the retrospective nature of this study and a limited sample size, these observations should be considered preliminary, and further prospective studies are needed for confirmation.

All patients had pulmonary involvement, which supports the respiratory tract as the primary route of NTM entry. All patients also had evidence of disseminated infection involving lymph nodes, the bone, skin, and pleura and accompanied by systemic inflammation. These features suggest that tracheobronchial NTM involvement may occur via hematogenous or lymphatic spread rather than via direct airway colonization. However, nonspecific presentations, which are often masked by systemic symptoms, make this manifestation easily overlooked. Definitive diagnosis requires histopathology and microbiological testing. In patients with endobronchial mass/nodule-like lesions—particularly those with risk factors for opportunistic infections—early microbiological evaluation is recommended to prevent complications such as airway stenosis. Respiratory specimens yielded the highest NTM isolation rates. Among the identified pathogens, 70% were slowly growing mycobacteria (e.g., *M. colombiense*, MAC), while rapidly growing mycobacteria (e.g., *M. abscessus*) were less common. The mNGS results were positive in all 11 patients tested. This analysis reduced the time to species identification by >1 week in 5 patients and detected NTM in 7 patients with negative conventional cultures, which suggests that mNGS is a sensitive method that may facilitate early diagnosis.

*M. colombiense* was first described in 2006 from Colombian lymphadenitis patients (Murcia et al., 2006). Our team previously characterized the clinical features of disseminated *M. colombiense* infection in an HIV-negative population (Tang et al., 2023). To our knowledge, this study provides the first systematic characterization of tracheobronchial involvement due to *M. colombiense* infection, which was observed in 7 patients. All 7 patients presented with disseminated NTM disease and mass/polypoid lesions, and bone destruction was observed in 6 (85.7%) of them. This phenotype—resembling invasive aspergillosis or malignancy—preliminarily suggests the presence of unique virulence factors that may warrant further genomic investigation.

Disseminated NTM disease has been reported in both immunocompetent individuals and patients with inborn errors of immunity. During the past decade, neutralizing anti-interferon-γ autoantibodies (nIFN-γ-autoAbs) have emerged as critical acquired immunodeficiencies that predispose adults to severe, disseminated NTM infection. These patients frequently harbor concomitant intracellular pathogens such as *Talaromyces marneffei*, Aspergillus spp., and nontyphoidal Salmonella, and the prevalence of nIFN-γ-autoAbs can reach 83.5% among HIV-negative adults with disseminated NTM disease (Qiu et al., 2022a). In the present study, 12 otherwise “healthy” patients presented with lymph node enlargement or osteolytic lesions together with disseminated tracheobronchial NTM infection; most patients also carried concurrent intracellular pathogens, including *T. marneffei* and Aspergillus. Although antibody testing was not performed in all patients, these clinical features raise the possibility of occult nIFN-γ autoAb-related immunodeficiency in a subset of this cohort. Patient 11, who was strongly positive for IFN-γ autoantibodies and had concomitant aspergillosis and *Pneumocystis jirovecii* infection, fulfilled the diagnostic criteria for adult-onset anti–IFN-γ autoantibody syndrome. Of note, Patient 18, who had no prior comorbidities, developed *T. marneffei* infection during his illness and ultimately died of respiratory failure and septic shock; this clinical course may also be consistent with underlying anti–IFN-γ autoantibody syndrome. Given these observations, clinicians may consider screening for anti–IFN-γ antibodies in adults with disseminated NTM infection, even in the presence of apparent immunocompetence, particularly when concomitant intracellular pathogens are present.

In patients with tracheobronchial NTM infection who have concomitant diseases, poor treatment response is frequently observed, and diagnostic delay is a core contributor to this clinical dilemma. In the present study, the median diagnostic delay was 130 days, reflecting substantial challenges in the clinical recognition of NTM infection. Furthermore, the radiological features of tracheobronchial NTM infection, including pulmonary masses, atelectasis, and mediastinal lymphadenopathy, along with bronchoscopic findings such as mass lesions, nodules, luminal narrowing, and purulent secretions (most commonly affecting the lobar bronchi), are difficult to distinguish from lung cancer, bronchial aspergillosis, or endobronchial tuberculosis.

Clinical practice indicates that differentiating NTM infection from lung cancer is extremely challenging, and the two conditions may coexist in the same patient, leading to **a** poor treatment response. A case of pulmonary MAC infection complicated by lung adenocarcinoma has been reported: the patient showed no clinical improvement after initial anti-MAC therapy and was ultimately diagnosed with adenocarcinoma by bronchoscopic pathology and cytology, suggesting that when response to standard anti-NTM treatment is suboptimal, concurrent malignancy should be strongly suspected (Mishra et al., 2024). Another study described a patient with imaging findings highly suspicious for lung cancer whose bronchial lavage fluid cultured positive for MAC; however, postoperative pathology confirmed the coexistence of adenocarcinoma and MAC within the same lesion (Taira et al., 2018). These findings indicate that in patients with a solitary pulmonary mass and positive mycobacterial culture from sputum or bronchial lavage fluid, the possibility of concurrent lung cancer cannot be readily excluded, even in the presence of microbiological evidence of NTM infection. However, in carefully selected patients with non-cavitary, minimally symptomatic disease, empiric anti-MAC therapy can lead to significant regression of suspected malignant nodules, thereby avoiding unnecessary surgery (Jacobs et al., 2026). Beyond malignancy, NTM co-infection with other pathogens also poses significant diagnostic and therapeutic challenges. The clinical manifestations of NTM and pulmonary TB overlap considerably, often leading to delayed diagnosis or misdiagnosis as either TB or NTM infection alone. In the present study, 65.5% of patients were initially misdiagnosed with pulmonary TB. It has been further reported that among patients with TB-NTM co-infection, 81.8% were diagnosed with NTM after TB, with a median interval of 5 months, and that co-infected patients had significantly worse survival outcomes than those with either infection alone (Fei et al., 2025). Another study reported a patient who was misdiagnosed with pulmonary TB for three years based solely on a positive acid-fast bacilli sputum smear, ultimately developing disseminated NTM and *T. marneffei* co-infection (Wu et al., 2025). This clinical course closely parallels the irreversible changes observed in our cohort, including airway stenosis and lung collapse, all resulting from diagnostic delay. Previously, we reported that elevated levels of anti-interferon-gamma autoantibodies, white blood cells, neutrophils, immunoglobulins (IgG, IgM, IgA), serum globulin, erythrocyte sedimentation rate, and C-reactive protein, as well as low CD4+ T cell counts, were risk factors for co-infection with *T. marneffei* and NTM (Qiu et al., 2021; Qiu et al., 2022b).

In summary, diagnostic delay in NTM infection is not merely a single event but rather a complex process closely related to host immune status, the type of co-infection, and underlying diseases. Whether facing the coexistence of NTM with lung cancer or co-infection with TB, fungi, or other mycobacteria, clinicians should proactively broaden the differential diagnosis when treatment response is poor. Molecular diagnostic techniques such as mNGS, along with repeated and multi-site pathogen and pathological testing, should be actively employed to establish an early etiological diagnosis and prevent irreversible organ damage.

The clinical courses of patient 2 and 19 suggest that even prolonged lung collapse with thoracic cage deformity may be reversible with appropriate medical and endoscopic therapy. In addition, diffuse tracheal nodules completely regressed in these patients. These observations raise that early initiation and completion of a full course of standardized therapy can effectively lead to remission and improvement of the lesions. Patient 16, who had no underlying disease, relapsed after 31 months, despite guideline-concordant, full-course treatment, which highlights the potential influence of host immunity on outcomes. Previous reports have indicated that relapse with the same species or reinfection with a different NTM strain after completion of therapy is not uncommon (Shin et al., 2018), suggesting that individualized treatment strategies and long-term surveillance may be beneficial. One published case described NTM infection extending from the larynx to the cervical trachea, culminating in subglottic stenosis (Lau et al., 2023). In our cohort, one patient developed airway obstruction secondary to endobronchial disease; their symptoms resolved only after combined anti-NTM chemotherapy and repeated bronchoscopy interventions. These findings suggest that early bronchoscopy may be considered for patients with tracheobronchial dissemination to assess the extent of airway involvement and that a combination of antimicrobial therapy and endoscopic intervention might improve outcomes. However, given the small sample size and retrospective design of this study, these observations should be considered preliminary, and prospective studies are needed to confirm the optimal timing and optimal approach to bronchoscopic intervention.

Our study employed stringent exclusion criteria to ensure phenotypic homogeneity, systematic bronchoscopic characterization, and longitudinal outcome data. The limitations include the small sample size—particularly for species-specific and multivariable analyses. The 29-patient cohort represents 10 years of consecutive enrollment; the effect sizes are large and consistent. The treatment heterogeneity (4 patients with HRZE empiricism) reflects real-world diagnostic evolution rather than protocol deviation, and sensitivity analyses support robustness.

Future directions include multicenter validation, whole-genome sequencing of *M. colombiense* to identify virulence determinants, prospective assessment of IFN-γ supplementation in AIGA, and long-term follow-up to define the optimal treatment duration and relapse predictors.

## Conclusions

Tracheobronchial NTM infection is rare but clinically significant and predominantly affects immunocompromised hosts. *M. colombiense* was the most common pathogen in our cohort. The diagnostic delay was substantial, with the condition frequently misdiagnosed as tuberculosis or malignancy. Bronchoscopy typically revealed mass/polypoid lesions and airway stenosis. In select cases, complete lung collapse may be reversible with targeted therapy. Early bronchoscopic evaluation, mNGS-based pathogen detection, and prompt antimicrobial therapy is essential to improve outcomes.

## Data Availability

The original contributions presented in the study are included in the article/**Supplementary Material**. Further inquiries can be directed to the corresponding authors.
