# Supplementary material for: Tracheobronchial invasion by nontuberculous mycobacteria: a rare but overlooked clinical manifestation—a multicenter retrospective analysis
Source: Front Cell Infect Microbiol. 2026 Jul 13;16:1872833. doi: 10.3389/fcimb.2026.1872833 (PMC13402115; doi:10.3389/fcimb.2026.1872833)
Supplement: Supplementary file 1 [file Table1.docx]

Table 1 Characteristics of the 29 patients with NTM infection of Tracheobronchial

| ID | Age/Sex | Comorbidity | Misdiagnosed diseases | Days required for diagnosis | Clinical manifestations | Chest CT | Fibreoptic bronchoscopy findings | Diagnostic methods | Site of infection | Mycobacterium species | NTM treatment | Outcome |
| --- | --- | --- | --- | --- | --- | --- | --- | --- | --- | --- | --- | --- |
| 1 | 42/M | None | Pulmonary Tuberculosis | 167 | Cough, expectoration, hemoptysis, low back pain, left shoulder pain, fatigue, anorexia, skin ulceration on the left upper limb | Nodules, linear opacities, consolidation, mass, mediastinal lymphadenopathy, destructive bone lesion at the right aspect of the T12 vertebra with an associated surrounding soft tissue mass. | Left bronchial mucosal hyperemia, Mass obstructing left upper lobe orifice with rough, uneven surfaces, Serous secretion | Sputum, BALF, Purulent secretions from left upper limb (c), pulmonary biopsy (h) | Bronchus, lung, skin, bone | NS | CAM, RFP, AMK, CFZ, LZD | Improved |
| 2 | 55/M | DM | Pulmonary Tuberculosis | 114 | Cough, expectoration, hemoptysis, left ear mass, weight loss | Patchy opacities, nodular opacities, linear opacities, consolidation, pleural thickening, bronchial stenosis, occlusion. | Distal trachea with multiple intraluminal neoplastic masses, left main bronchus demonstrating mucosal edema, right lateral wall covered by granulomatous neoplasm, and distal segment stenosis, left upper lobe orifice completely occluded, Serous secretions present | BALF(c), sputum (c+ mNGS) | Trachea-bronchus, lung, skin | Mycobacterium colombiense | CAM, RBT, EB, INH | Improved |
| 3 | 42/F | AIDS | Pulmonary Tuberculosis | 44 | Cough, expectoration, cervical lymphadenopathy | Patchy opacities, nodules, linear opacities, and mediastinal lymphadenopathy. | Bilateral bronchial mucosal hyperemia and edema, luminal obstruction by a mass in the left lower lobe dorsal segment, intraluminal protruding mass with localized gray-black necrotic material extending from the distal right intermediate bronchus to the right lower lobe dorsal segment, luminal obstruction by masses in both the left and right lower lobe dorsal segments, serous secretions | BALF, sputum (c+PCR) | Bronchus, lung, lymph node | *M. avium* | CAM, RBT, EB, MFLX | Improved |
| 4 | 46/M | AIDS | None | 51 | Fever, cough, expectoration, chest pain | Patchy opacities, nodules, cavity, bronchial stenosis/occlusion, pleural effusion, and mediastinal lymphadenopathy. | Left upper lobe and right upper lobe bronchial mucosa with hyperemia, edema, and varying degrees of hyperplasia, complete occlusion of the left upper lobe anterior segment orifice, stenosis of the left upper lobe apical-posterior segment orifice and all right upper lobe segmental orifices, mass with surface necrosis located at the left upper lobe orifice, viscous intraluminal secretions present | BALF (mNGS), Blood(c) | Bronchus, lung, lymph node | *M. avium* | AZM, RFP, EB, AMK | Improved |
| 5 | 31/M | AIDS | Pulmonary Tuberculosis | 511 | Cough, cervical lymphadenopathy | Patchy opacities, nodules, atelectasis, bronchial occlusion, pericardial effusion, mediastinal lymphadenopathy. | Right middle and lower lobe bronchial mucosa with hyperemia and edema, right middle lobe orifice completely obstructed by an intraluminal mass | BALF, sputum (c+PCR) | Bronchus, Lung, Lymph Node | *M. avium* | HRZE | Improved |
| 6 | 54/F | DM, Erythema Nodosum | Pulmonary Tuberculosis | 54 | Cough, expectoration, hemoptysis, abscess formation in the right inguinal and submental regions | Patchy opacities, linear opacities, bilateral pleural thickening, bilateral pleural effusion, hilar and mediastinal lymphadenopathy, rib destruction. | Purulent secretions in the left main bronchus, luminal mass with mucosal hyperemia and edema in the right intermediate bronchus | sputum (c+PCR), Skin pus (PCR+mNGS) | Trachea, Lung, Skin, Lymph Node, Rib | Mycobacterium colombiense | CAM, RFP, EB, MFLX | Improved |
| 7 | 43/M | Bladder cancer with surgery and chemotherapy, Hepatitis B carrier | Pulmonary Tuberculosis | 233 | Fever, cough, expectoration, chest pain, shortness of breath, fatigue, weight loss, cervical lymphadenopathy | Patchy opacities, nodules, linear opacities, pleural thickening, mediastinal lymphadenopathy. | A granulomatous neoplasm was observed in the left main bronchus | BALF (mNGS) | Trachea, Lung, Right Pleura, Cervical Lymph Node | Mycobacterium florentinum | CFX, EB, MFLX, LZD | Improved |
| 8 | 55/M | None | Pulmonary Tuberculosis | 207 | Cough, expectoration, hemoptysis, chest pain | Prominent and disorganized lung markings, linear opacities, consolidation, bronchial stenosis/occlusion, pleural effusion, and destructive bone lesions of the C6-T2, T10 vertebra with surrounding soft tissue swelling. | Left upper lobe bronchial mucosa with hyperemia and edema, granulomas of varying sizes protruding from the mucosa above the carina and at the orifices of the left upper and lower lobes, a friable, easily bleeding mass completely obstructing the left upper lobe orifice, stenosis of the left upper lobe orifice | Sputum(c), bronchial mucosal tissue (h) | Bronchus, Lung, Cervicothoracic Vertebral Body | NS | CAM, AMC/CVA, EB, Pto | Improved |
| 9 | 66/F | Pulmonary Tuberculosis | Pulmonary Tuberculosis | 19 | Fever, cough, expectoration | Prominent and disorganized lung markings, nodules, linear opacities. | A few scattered small nodules visible in the mucosa of the distal left main bronchus. | Sputum, +blood (c), BALF (c+mNGS) | Trachea, Lung | *M. intracellulare* | CAM, RFP, EB | Improved |
| 10 | 44/F | None | Pulmonary Tuberculosis | 130 | Cough, expectoration, hemoptysis, fatigue, weight loss, joint pain, swollen lymph nodes, rash | Nodules, linear opacities, mediastinal lymphadenopathy. | Two masses with brownish surfaces were visible in the right main bronchus. | BALF (mNGS), supraclavicular lymph node biopsy (h) | Trachea, Lung, Skin, Lymph Node | *M. abscessus* | CAM, CFX, TGC, AMK, LZD | Improved |
| 11 | 40/M | Anti-IFN-γ autoantibodyassociated immunodeficiency syndrome | Pulmonary Tuberculosis | 141 | Cough, expectoration, skin lesion | Patchy opacities, nodules, consolidation, bronchial stenosis, mediastinal lymphadenopathy. | Left main and left upper lobe bronchial mucosa with hyperemia and edema, localized mucosal thickening with irregular, granular surface texture, friable on contact, varying degrees of stenosis in the left upper lobe lumen. Serous secretions. | Sputum, left forearm skin lesion(c) | Trachea/Bronchus, Lung, Skin, Lymph Node | Mycobacterium colombiense | CAM, RFP, CFX, EB, AMK | Improved |
| 12 | 30/F | None | Pulmonary Tuberculosis and Tuberculous Lymphadenitis | 502 | Fever, cough, expectoration, headache, fatigue, weight loss, swollen lymph nodes, skin lesion | Consolidation, pleural thickening, bronchial stenosis. | Left main bronchus and orifices of left upper and lower lobes showed multiple nodular protrusions with thickened, coarse mucosa, luminal stenosis at the left lower lobe orifice, purulent secretions | BALF (mNGS) | Trachea/Bronchus, Lung, Skin, Lymph Node | NS | CAM, RFP, INH, EB, MFLX | Non-improvement |
| 13 | 65/F | Hypothyroidism | Lung Cancer | 43 | Fever, cough, expectoration, fatigue, anorexia, shortness of breath | Patchy opacities, consolidation, pleural thickening, bronchial stenosis, pleural effusion, mediastinal lymphadenopathy. | Scattered ulcers of varying sizes on the right lateral tracheal wall, irregular luminal stenosis in the right upper lobe bronchus, diffusely growing mass with irregular surface on the bronchial wall | BALF (mNGS) | Trachea/Bronchus, Lung, Lymph Node | *M. colombiense* | CAM, RFP, EB, MFLX | Non-improvement |
| 14 | 54/M | AIDS | Lung Cancer, Tuberculous Lymphadenitis | 50 | Fever, chills, cough, expectoration, headache, fatigue, anorexia, weight loss, swollen lymph nodes | Patchy opacities, nodules, linear opacities, consolidation, pleural thickening, pleural effusion, pericardial effusion, mediastinal lymphadenopathy. | Bilateral bronchial mucosal hyperemia and edema, thickened longitudinal mucosal folds causing luminal compression, nodular protrusion on left main bronchial mucosa, stenosis of left upper lobe, right upper lobe, and right middle lobe orifices, serous secretions | BALF (mNGS) | Bronchus, Lung, Lymph Node, Pleura | *M. abscessus* | AZM, RBT, EB | Improved |
| 15 | 37/F | None | Pulmonary Tuberculosis | 90 | Fever, cough, expectoration, headache, chest pain, weight loss, skin lesion | Prominent and disorganized lung markings, patchy opacities, nodules, linear opacities, mediastinal lymphadenopathy. | Left and right bronchial mucosa with hyperemia and edema, multiple nodular neoplastic growths in the mucosa of the left lower lobe basal segments, serous secretions. | Sputum, BALF, skin lesion (c) | Bronchus, Lung, Lymph Node, Skin | *M. avium* | INH, RFP, EB, AMK, MFLX | Improved |
| 16 | 39/M | None | Lung Cancer | 239 | Cough, expectoration, hemoptysis, back abscess, swollen lymph nodes | Prominent and disorganized lung markings, patchy opacities, bronchial stenosis/occlusion, mediastinal lymphadenopathy, destructive bone lesion of the T11 vertebra. | Carina and bilateral bronchial orifices with hyperemia, edema, and thickening, friable on contact, irregular mass at the carina extending proximally into both main bronchi, right main bronchial stenosis, purulent secretions. | BALF(c+mNGS), Back skin puncture (c+h) | Bronchus, Lung, Lymph Node, Skin, bone | *M. avium- complex* | CAM, CFX, AMK | Recurrence |
| 17 | 53/F | None | Pulmonary Tuberculosis | 133 | Fever, cough, swollen lymph nodes, skin lesion | Prominent and disorganized lung markings, patchy opacities, nodules, linear opacities, pleural effusion, mediastinal lymphadenopathy, bone destruction. | Scar formation at the left main bronchial orifice, thickened and irregular mucosa at the right lower lobe basal segment orifice | Bone marrow(c) | Bronchus, Lung, Lymph Node, Bone, Bone Marrow, Skin | NS | CAM, INH, EB, PZA | Died |
| 18 | 50/F | None | None | 24 | Fever, cough, expectoration, shortness of breath, low back pain | Prominent and disorganized lung markings, patchy opacities, nodules, linear opacities, multiple osteolytic bone lesions | Bilateral bronchial mucosa with hyperemia and edema, thickened mucosal folds in the right lower lobe bronchus with scant grayish-white deposits, friable on contact, stenosis of the right lower lobe bronchus, purulent secretions. | BALF (mNGS) | Bronchus, Lung, Lymph Node, Bone | *M. intracellulare* | AZM, INH, RFP, EB, MFLX | Cure |
| 19 | 48/F | None | Pulmonary Tuberculosis | 929 | Cough, expectoration, hemoptysis, fatigue, anorexia, swollen lymph nodes, skin lesion | Patchy opacities, nodules, linear opacities, consolidation, mass, pleural thickening, pericardial and pleural effusion, bronchial stenosis, bone destruction. | Left main bronchial mucosa with hyperemia and edema, irregular bronchial wall surface covered by yellowish-white necrotic material, neoplasm completely obstructing the left main bronchial lumen | BALF (c+mNGS), Sputum, tracheal mass tissue (c) | Trachea, Lung, Lymph Node, Skin, bone | *M. avium complex* and *M. colombiense* coinfection | CAM, RFP, EB, CFZ, Bronchoscopic interventional therapy | Improved |
| 20 | 47/M | Gastritis | Lung Cancer | 264 | Fever, cough, expectoration, hemoptysis, shortness of breath, fatigue, anorexia, low back pain，weight loss | patchy opacities, nodules, pleural thickening, mass, bronchial stenosis, and bone destruction involving the L1-L2 vertebrae, sternum, and multiple thoracic vertebrae. | Upper tracheal wall with nodular neoplastic growth, bilateral bronchial mucosa with hyperemia and edema, serous secretions | Pulmonary biopsy (c+h) | Trachea, Lung, bone | *M. colombiense* | CAM, RFP, EB, AMK | Cured |
| 21 | 57/F | Pulmonary Tuberculosis | Lung Cancer | 301 | Cough, expectoration, swollen lymph nodes | Patchy opacities, nodules, mass, bronchial stenosis, pleural effusion, hilar and mediastinal lymphadenopathy, bone destruction involving ribs, thoracic vertebrae, sternum, humerus, and scapula | Upper tracheal wall with nodular neoplastic growth, bilateral bronchial mucosa with hyperemia and edema, serous secretions | BALF (c+mNGS) | Bronchus, Lung, Bone, Lymph Node | *M. intracellulare* | CAM, RFP, EB, AMK | Improved |
| 22 | 44/M | Status post left lower lobectomy | Pulmonary Tuberculosis | 173 | Cough, expectoration, fatigue, night sweats | Patchy opacities, nodules, cavity, pleural thickening, pericardial and pleural effusion, mediastinal lymphadenopathy, bone destruction involving T9-T10 vertebrae, manubrium sterni, and ribs. | Stenosis of the apical-posterior segmental bronchus of the right upper lobe with mucosal hyperemia, posterior segmental bronchus obstructed by mucosal protrusion, eroded and elevated mucosa on the lateral wall of the right upper lobe orifice, friable on contact | BALF(c+G) | Lung, Bronchus, Bone, Lymph Node | *M. avium* | CAM, INH, EB, AMK, MFLX | Improved |
| 23 | 38/M | AIDS | Pulmonary Tuberculosis | 98 | Fever, cough, shortness of breath, fatigue, swollen lymph nodes | Patchy opacities, nodules, consolidation. | A granulomatous neoplasm with surface necrosis and hemorrhagic tendency was identified on the anteromedial wall of the mid-portion of the left main bronchus. | BALF(c) | Lung, Bronchus, Lymph Node | NS | Not on anti-NTM treatment | Non-improvement |
| 24 | 50/M | None | Pulmonary Tuberculosis | 415 | Cough, expectoration, shortness of breath, swollen lymph nodes | Nodules, pericardial effusion, pleural effusion, mediastinal lymphadenopathy. | Significant stenosis of the right intermediate bronchus with local mucosal scarring, diffuse granulomatous changes and focal mucosal erosion on the medial wall of the right main bronchus | Skin pus, BALF(c) | Lung, Bronchus, Lymph Node | NS | HRZE to anti-TB treatment, Not on anti-NTM treatment | Non-improvement |
| 25 | 60/M | COPD | None | 738 | Cough, expectoration, shortness of breath | Patchy opacities | Marked stenosis of the right intermediate bronchus with focal mucosal scarring, diffuse granulomatous transformation with focal mucosal erosion on the medial wall of the right main bronchus | BALF (mNGS) | Lung, Bronchus | Mycobacterium chelonae | Not on anti-NTM treatment | Improved |
| 26 | 70/M | Chemotherapy for lung malignancy | None | 7 | Cough, expectoration, hemoptysis, shortness of breath, fatigue, anorexia, swollen lymph nodes | Patchy opacities, nodules, and bronchial wall thickening. | A neoplastic mass with irregular, hyperemic, and hemorrhagic surface completely obstructs the orifice of the left lower lobe basal segment bronchus. | Sputum (c+G) | Lung, Bronchus, Lymph Node | *M. intracellulare* | HRZE to anti-TB treatment, Not on anti-NTM treatment | Non-improvement |
| 27 | 47/F | Pulmonary Tuberculosis | None | 39 | Cough, expectoration, fatigue, anorexia, arthralgia and swelling with restricted mobility, swollen lymph nodes | Patchy opacities, nodules, linear opacities, bronchiectasis, pleural thickening, pleural effusion, cavity, bone destruction involving the ribs, sternum, and thoracolumbar spine. | Small nodular mucosal protrusions in the left main and left lower bronchi, stenosis of the right lower lobe bronchus, small hemorrhagic nodular mucosal protrusions in the right main, right upper, right intermediate, and right lower bronchi | Skin pus (mNGS) | Lung, Bronchus, Lymph Node, Skin, bone | *M. avium* and *M. colombiense* coinfection | CAM, RBT, EB, AMK | Improved |
| 28 | 51/M | None | Pulmonary Tuberculosis | 11 | Cough, expectoration, swollen lymph nodes | Patchy opacities, nodules, bronchial stenosis, mediastinal lymphadenopathy. | Grayish-black granulomatous protrusion at the right upper lobe bronchial orifice, partially obstructing the right anterior segmental orifice, with mucosal hyperemia and thickening, mucosal scar-like changes in the right intermediate bronchus | BALF (c+G) | Lung, Bronchus, Lymph Node | *M. kansasii* | CAM, RFP, EB, AMK | Improved |
| 29 | 45/M | None | Pulmonary Tuberculosis | 36 | Fever, cough, expectoration, shortness of breath, fatigue, swollen lymph nodes | Patchy opacities, nodules, bronchiectasis, pleural thickening, pericardial effusion, pleural effusion. | Mucosal hyperemia, neoplastic mucosal growth in the right main bronchus, right intermediate bronchial orifice, and right upper lobe anterior segmental bronchus | Transbronchial biopsy (mNGS) | Lung, Bronchus, Lymph Node | *M. intracellulare* | HRZE to anti-TB treatment, Not on anti-NTM treatment | Improved |

Diagnostic methods to demonstrate NTM infection were culture (c), histopathology (h), metagenomic next-generation sequencing (mNGS), PCR and Gene microarray method (G)

Abbreviations: NTM, nontuberculous mycobacteria; BALF, bronchoalveolar lavage fluid;

AMK, amikacin; RFP, rifampicin; AZM, azithromycin; CAM, clarithromycin; CFZ，Clofazimine；LZD, linezolid; INH, isoniazid; EB, ethambutol; RBT, rifabutin; MFLX, moxiffoxacin; CFX, Cefoxitin; AMC/CVA, Amoxicillin/Clavulanate; Pto, Protionamide; TGC, Tigecycline; PZA, Pyrazinamide

DM, Diabetes Mellitus

NS
